# Depression and coronary heart disease: mechanisms, interventions, and treatments

**DOI:** 10.3389/fpsyt.2024.1328048

**Published:** 2024-02-09

**Authors:** Linjie Xu, Xu Zhai, Dazhuo Shi, Ying Zhang

**Affiliations:** ^1^ National Clinical Research Center for Chinese Medicine Cardiology, Xiyuan Hospital of China Academy of Chinese Medical Sciences, Beijing, China; ^2^ Graduate School of Beijing University of Chinese Medicine, Beijing, China; ^3^ Wangjing Hospital, China Academy of Chinese Medical Sciences, Beijing, China

**Keywords:** coronary heart disease, depression, bipolar heart disease, mechanisms, antidepressant treatment

## Abstract

Coronary heart disease (CHD), a cardiovascular condition that poses a significant threat to human health and life, has imposed a substantial economic burden on the world. However, in contrast to conventional risk factors, depression emerges as a novel and independent risk factor for CHD. This condition impacts the onset and progression of CHD and elevates the risk of adverse cardiovascular prognostic events in those already affected by CHD. As a result, depression has garnered increasing global attention. Despite this growing awareness, the specific mechanisms through which depression contributes to the development of CHD remain unclear. Existing research suggests that depression primarily influences the inflammatory response, Hypothalamic-pituitary-adrenocortical axis (HPA) and Autonomic Nervous System (ANS) dysfunction, platelet activation, endothelial dysfunction, lipid metabolism disorders, and genetics, all of which play pivotal roles in CHD development. Furthermore, the effectiveness and safety of antidepressant treatment in CHD patients with comorbid depression and its potential impact on the prognosis of CHD patients have become subjects of controversy. Further investigation is warranted to address these unresolved questions.

## 1 Introduction

Coronary Heart Disease (CHD), a significant health threat, is steadily increasing yearly, often accompanied by varying mood disorders. Depression, currently the second leading cause of death worldwide after CHD itself, has emerged as an independent risk factor apart from traditional CHD risk factors such as hypertension, hyperlipidemia, diabetes, smoking, and obesity (1). The coexistence of CHD and depression significantly adds to the global healthcare burden (2).

Epidemiological surveys indicate that approximately one-fifth of Americans will experience depression at some point (3). Moreover, the prevalence of depression among CHD patients is exceptionally high, ranging from 15% to 30%, significantly surpassing the 10% prevalence in the general population (4). International studies involving 30 countries and regions reveal an overall prevalence, 1-year prevalence, and lifetime prevalence of depression to be 12.9%, 7.2%, and 10.8%, respectively (5).

Existing research on CHD comorbid with depression primarily relies on depressive symptom scales and often exhibits a dose-response pattern (6, 7). This means that higher levels of depressive symptoms are associated with a greater risk of developing CHD. Nevertheless, causal associations between depression and CHD remain controversial. The physiological pathways connecting depression to CHD are notably intricate, making an in-depth understanding of this relationship crucial for developing rational and effective interventions in clinical treatment. Such efforts are essential to enhance patients’ quality of life and improve cardiovascular outcomes.

This article aims to summarize the effects of depression on CHD and evaluate the efficacy of antidepressant therapy, in order to provide valuable insights and a foundation for clinical treatment strategies and the development of new drugs.

## 2 Depression as a risk factor for CHD

Depression exhibits a high comorbidity with CHD, and its association with CHD is even stronger when compared to traditional risk factors for cardiovascular disease (8). This mood disorder increases the risk of developing CHD (9) and significantly impacts prognosis (10). A 1-year follow-up study involving 1024 CHD patients revealed that depression constituted a significant risk factor for mortality in this group (HR = 3.19, 95% CI 1.32-7.69). Moreover, the severity of the depressive state correlated with a worse prognosis and a higher incidence of adverse cardiovascular outcomes (11).

At the outset of the 21st century, a comprehensive international study by 30 health organizations examined the interrelationship between psychosomatic factors and CHD. It revealed that, even after accounting for traditional risk factors such as hypertension and obesity, the incidence of CHD was three times higher in individuals with depression (12). In a substantial cohort study involving 1.9 million participants, depression significantly elevated the occurrence of stable/unstable angina and myocardial infarction in CHD patients (13). However, conflicting perspectives exist regarding whether depression increases morbidity and mortality in CHD patients. For instance, the ENRICHD study, a well-known clinical trial in the CHD field, reported that depression did not increase morbidity or mortality, precisely due to CHD. Still, it did lead to an overall increase in mortality (14). Moreover, the impact of depression on CHD appears to be influenced by factors such as gender, age, and the duration of the illness. A Meta-analysis indicated that depression did not raise the risk of CHD in patients with more than 15 years of follow-up. However, it did correlate with an increased incidence of CHD in patients with less than 15 years of follow-up (15). Further reinforcing the association, a 3-year follow-up study confirmed that CHD patients with comorbid depression had a significantly higher morbidity and mortality rate than those with CHD alone (16).

Furthermore, depression can undermine treatment adherence in CHD patients, posing an additional challenge to their healthcare. A study utilizing a medication monitor to gather objective data on patient medication adherence discovered that CHD patients with comorbid depression displayed lower adherence to low-dose aspirin, evident from reduced drug concentrations. Gehi et al. (17) assessed medication adherence through self-reporting in a cohort of 940 patients discharged from the hospital with stable CHD. The findings revealed that the nonadherence rate was 5% among CHD patients without depression, 7% in those with moderate depression, and 14% in those with major depression. In summary, depression impacts the morbidity of CHD and exerts an exceptionally negative influence on its treatment and prognosis. Even with the most well-designed secondary prevention program for CHD, depression can still precipitate adverse cardiovascular events in patients with CHD. As a result, clinicians should be vigilant in screening for depression among CHD patients, and timely provision of antidepressant treatment becomes paramount to improving patient outcomes and reducing morbidity and mortality rates.

## 3 Mechanisms

**Figure 1:**
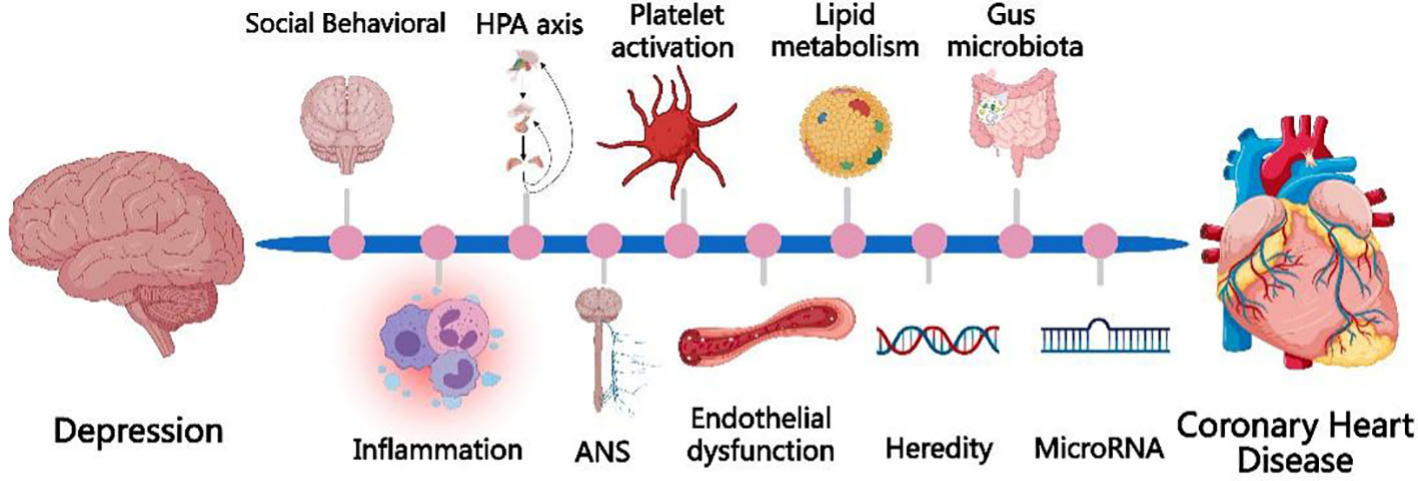
The currently proposed mechanisms of depression causing CHD include socio-behavioral mechanisms and physiological mechanisms, such as over-activation of the inflammatory response, autonomic dysfunction, endothelial dysfunction, abnormal HPA axis function, platelet over-activation, and genetics, microRNA, gut microbiota.

There are many hypotheses about the possible mechanisms by which depression contributes to the development of CHD, but they are still not fully elucidated. The currently proposed mechanisms of depression causing CHD include socio-behavioral and physiological mechanisms such as over-activation of the inflammatory response, autonomic dysfunction, endothelial dysfunction, abnormal HPA axis function, platelet over-activation, and genetics. The mechanism is shown schematically in **Figure 1**.

### 3.1 Social behavioral and type D personality

Behavioral abnormalities in depressed patients may indeed contribute to the development of CHD. In today’s fast-paced lifestyle, characterized by tremendous work, economic, and social stress, the silent toll it takes on human physical and mental health is alarming. Depressed individuals often experience low mood, loss of interest in their surroundings, a significant reduction in physical activity compared to healthier individuals, and may even have suicidal tendencies. A study conducted in 2011 that assessed the depressive status and long-term follow-up of 5,888 older adults discovered that behavioral activities, such as physical activity and depression, were independent predictors of cardiovascular disease mortality. Importantly, the association between depression and cardiovascular disease was strong (18). Chronic stress emerges as a significant risk factor for CHD, leading to an increased susceptibility to cardiovascular events. Chronic stress contributes to this heightened risk by activating the Hypothalamic-Pituitary-Adrenal (HPA) axis, causing autonomic dysfunction and promoting inflammatory responses (19). Studies have consistently demonstrated the link between chronic stress and an increased incidence of depression. Prolonged stress triggers changes in neurotransmitters and neuroendocrine molecules, affecting neuronal activity and potentially contributing to the development of depression through activating the HPA axis and the elevation of pro-inflammatory factors (20).

Type D personality, also known as “sad personality,” refers to an individual’s chronic experience of negative emotions, such as sadness, anxiety, irritability, and anger, as well as a tendency to express negative emotions in social situations. The main personality traits of Type D individuals are negative emotions and social inhibition. CHD patients with a Type D personality are more prone to experiencing depression, and the severity of their Type D personality traits is positively correlated with depression (21). Furthermore, Lin et al. (21) demonstrated that Type D personality is associated with an increased risk of adverse cardiovascular events and death in patients with CHD. The pathophysiological mechanisms underlying this association may involve dysfunction of the cardiac Autonomic Nervous System (ANS) and dysregulation of lipid profiles.

Furthermore, CHD patients who suffer from depression may exhibit decreased treatment adherence, failure to take medications as prescribed, delayed participation in cardiac rehabilitation programs, and some may even become overweight or obese due to side effects of tricyclic antidepressants. Depressed patients are more prone to engaging in adverse lifestyle behaviors such as smoking and alcohol abuse (22). These behavioral abnormalities contribute to an increased incidence of CHD and result in a significant rise in adverse cardiovascular events. Therefore, these behavioral abnormalities play a role in the likelihood of developing CHD and contribute to a significant increase in adverse cardiovascular events.

### 3.2 Inflammatory response

In the late 1990s, CHD was identified as an inflammatory disorder, and subsequent studies have demonstrated the crucial role of the inflammatory response in the atherosclerotic process (23). Moreover, depression has been linked to a persistent inflammatory state closely associated with the inflammatory response. The inflammatory response is a significant mechanism in the pathogenesis of depression and CHD, and it may be a key factor in how depression contributes to the development of CHD (22). In individuals with depression, the risk of the body being stimulated by bacterial lipopolysaccharide increases, leading to heightened secretion of cytokines by monocytes. This results in the overexpression of inflammatory factors and cytokines like C-reactive protein, tumor necrosis factor, and interleukins. The degree of elevation of these cytokines is positively correlated with the severity of depression (24). These cytokines, individually or in synergy, prompt the conversion of monocytes into pro-inflammatory cells, mediating non-specific inflammatory response and altering immunoreactivity. Additionally, these cytokines can impact the cardiovascular system by influencing the release of neurotransmitters from the central nervous system, thereby mediating sympathetic effects (25). Depression and inflammation exhibit a bidirectional relationship: depression can promote an inflammatory response, and conversely, blocking pro-inflammatory factors (e.g., TNF-α) can partially alleviate depressive symptoms (26). Apart from increased pro-inflammatory factors, a decrease in anti-inflammatory factors has also been associated with depression. For instance, Li et al. (27), in their study on the relationship between depression and inflammatory factors in cancer patients, found a negative correlation between depression severity and plasma levels of the anti-inflammatory factor IL-4. These findings suggest that depression may involve up regulation of pro-inflammatory factors and down regulation of anti-inflammatory factors.

The inflammatory response induced by depression is closely associated with the development of CHD and adverse cardiovascular events. Specifically, CRP, IL-6, and TNF-α can destabilize atherosclerotic plaques, leading to plaque rupture and thrombosis at a later stage. Additionally, they directly affect myocardial contractility, inducing cell apoptosis (28). Experimental studies have shown that chronic stress increases macrophage infiltration and atherosclerotic plaque growth and vulnerability in a mouse model, which is associated with the development of myocardial infarction and injury (29). Clinical studies have found a positive correlation between high levels of hs-CRP and the severity of depression at baseline in patients with myocardial infarction (30). Empana et al. (31), in a prospective epidemiologic study of European patients with myocardial infarction, observed that Welsh Depression Scale scores were associated with elevated levels of IL-6 (OR 1.39), CRP (OR 1.30), intercellular adhesion molecule 1 (OR 1.22), and plasma fibrinogen (OR 1.36) after correcting for other risk factors compared to age-matched controls with angina pectoris or nonfatal myocardial infarction. Chronic inflammation can cause damage to arterial walls and increased activity of T and B lymphocytes, suggesting that depression may contribute to or worsen CHD through the inflammatory response process (32). In conclusion, inflammation and immunity represent significant and promising research areas in studying the pathogenesis of CHD related to depression. In depressed patients, increased pro-inflammatory and decreased anti-inflammatory factors may be associated with CHD’s onset, progression, and prognosis. As more inflammatory markers are used to study CHD and depression, the mechanism of the inflammatory response’s involvement in depression leading to CHD will be further elucidated in depth.

### 3.3 Dysfunction of the HPA axis

HPA axis dysfunction has been extensively studied in the context of depression. Elevated circulatory cortisol levels, resulting from enhanced HPA axis activity due to depression, may represent another possible biological mechanism that triggers CHD (33). In patients with depression, the HPA axis is activated, and the paraventricular nucleus of the hypothalamus synthesizes and releases corticotropin-releasing hormone (CRH) and arginine vasopressin, which are then transported to the anterior pituitary gland and stimulate the secretion of adrenocorticotropic hormone (ACTH) into the systemic circulation. Subsequently, ACTH acts on the zona fasciculata of the adrenal cortex, promoting cortisol synthesis and release. CRH, in turn, increases heart rate, cardiac output, and mean arterial pressure by stimulating the secretion of norepinephrine and epinephrine. Cortisol influences the cardiovascular system, increasing myocardial contractility, cardiac output, and blood pressure. It also contributes to the redistribution of visceral fat, triggers inflammatory responses, leads to insulin resistance, and results in hypercholesterolemia – all risk factors for CHD. Moreover, cortisol plays a role in inhibiting growth hormone secretion and activity of the gonadal axis (34). Deficiency in growth hormone increases the risk of adverse cardiovascular events, while testosterone deficiency is associated with metabolic abnormalities and poor prognosis in patients with cardiovascular disease (35). On the other hand, estrogen’s protective effects on the cardiovascular system have long been recognized. In summary, HPA axis dysfunction and the subsequent elevation of cortisol levels can contribute to the development of CHD, potentially through various cardiovascular and metabolic effects. Additionally, the interplay of growth, testosterone, and estrogen hormones further influences cardiovascular health and outcomes in patients with CHD and depression.

Indeed, depression can lead to changes in neuroendocrine function, activating both the HPA axis and the sympathetic nervous system. This activation, in turn, promotes the release of angiotensin-II and glucocorticoids, subsequently activating the glucocorticoid receptor, thereby increasing the risk of CHD (36). Studies on rat models of depression have shown significantly elevated levels of glucocorticoids, which were found to reduce the viability of cardiomyocytes cultured *in vitro* (37). Glucocorticoids can directly impact the pathophysiological mechanisms of the cardiovascular system. They can lead to the over-activation of sympathetic nerves, elevate blood pressure, increase myocardial oxygen consumption, and enhance the sensitivity of the cardiovascular system to catecholamines. These effects can contribute to an increased risk of CHD (37). In summary, depression-induced changes in neuroendocrine function, particularly the activation of the HPA axis and sympathetic nervous system, can elevate glucocorticoid levels, affecting the cardiovascular system and increasing susceptibility to CHD. The intricate interplay of these mechanisms highlights the complex relationship between depression and cardiovascular health.

### 3.4 Autonomic nervous system dysfunction

The relationship between ANS (Autonomic Nervous System) dysfunction, depression, and CHD has been extensively studied, and impaired ANS function is considered an intermediate risk factor that links depression to an increased risk of CHD. As mentioned earlier, depression can lead to the hyperfunction of the HPA axis, which, in turn, can alter cardiac autonomic function through central nervous system regulation. This alteration results in sympathetic overactivation and parasympathetic hypofunction. Sympathetic overactivation can have various detrimental effects on the cardiovascular system. On one hand, it can lead to the excitation of α-receptors, causing coronary artery spasm and vasoconstriction, resulting in myocardial ischemia and increased blood pressure. On the other hand, sympathetic overactivation can stimulate β-receptors, increasing heart rate, myocardial contractility, and myocardial oxygen consumption. This effect lowers the threshold for ventricular fibrillation, making the heart more susceptible to ventricular tachycardia, ventricular fibrillation, and other malignant cardiac arrhythmias. Additionally, it increases the risk of sudden cardiac death (38).

Heart rate variability (HRV) is an important repeatable quantitative and noninvasive indicator of cardiac autonomic nervous system (ANS) function (39). It refers to the variation in sinus rhythm (the standard deviation of the intervals between two consecutive R-waves in sinus rhythm on the electrocardiogram) over a certain period, typically 24 hours. HRV reflects the ability of the ANS to change heart rate in response to changes in hemodynamics or other physiological disturbances, indicating the interaction and balance between sympathetic and parasympathetic nerves in regulating heart rate. A decrease in HRV suggests dysfunction of the cardiac ANS, which is considered a strong and independent predictor of poor prognosis in CHD. Notably, HRV has also been a focus of biomarker studies in depression. Research has found that HRV is significantly lower in patients with major depressive disorder than in healthy controls and tends to normalize as depressive symptoms improve. Various parameters of HRV, such as the standard deviation of all sinus beat R-R intervals (SDNN), root mean square of the difference between neighboring R-R intervals (rMSSD), and the percentage of R-R intervals with a difference of more than 50 ms to the total number of R-R intervals (PNN50), are reduced in depression (40, 41). Depression can lead to sympathetic excitation, resulting in an increased heart rate in patients, and a higher incidence of various tachyarrhythmias compared to CHD patients without comorbid depression (42). Individuals with CHD and depression exhibit lower HRV, reduced pressure receptor sensitivity, increased QT interval variability, and heart rate oscillations compared to those without depression (43). Animal experiments have also confirmed that depression significantly increases the incidence of ventricular arrhythmias, prolongs the QT interval, increases sympathetic excitability, and enhances post-infarction myocardial remodeling in rats (44). These findings collectively suggest that depression can adversely affect CHD by influencing ANS function and, consequently, impacting HRV and CHD outcomes. However, whether depression affects HRV in all types of patients with CHD remains controversial. Some studies have shown that depression causes a significant decrease in HRV and a significant increase in heart rate in patients with stable angina (45). In contrast, other studies have found that depression has no significant effect on HRV in patients with unstable angina (46). Furthermore, it has been suggested that HRV reduction may be associated only with specific depressive symptoms (47). Nonetheless, some evidence indicates that antidepressants can significantly affect HRV (48). These discrepancies may stem from the complexity of depression and its various interactions with ANS function in different populations of CHD patients.

After excessive activation of sympathetic nerves due to depression, catecholamine levels are elevated, and adrenergic receptor action is enhanced, significantly increasing the risk of adverse outcomes such as the development of CHD and malignant arrhythmias, and even sudden cardiac death. Experimental studies in rats with depression combined with Myocardial Infarction (MI) have shown elevated plasma levels of epinephrine and norepinephrine, along with higher levels of myocardial tyrosine hydroxylase expression compared to the MI-only group. Additionally, these rats exhibited increased left ventricular volume and thinned myocardial walls, suggesting that depression may contribute to the unfavorable prognosis of MI by enhancing sympathetic nerve activity and promoting ventricular remodeling (49). Hence, it is evident that the hyperfunction of the HPA axis induced by depression, coupled with the overactivation of sympathetic nerves, collaborates to reduce myocardial stress tolerance, significantly influencing the pathogenesis and prognosis of CHD.

### 3.5 Platelet activation and 5-hydroxytryptamine

The primary pathological change in CHD is atherosclerosis, characterized by an inflammatory process that involves impaired endothelial function mediated by platelets and leukocytes. Depression plays a role in promoting CHD by activating platelets through sympathetic overactivation and influencing the 5-HT (serotonin) system, which can lead to the rupture of atherosclerotic plaques and thrombosis.

Ormonde do Carmo et al. (50) conducted a study comparing blood samples from 22 depressed patients without antidepressant treatment to 27 healthy controls. The findings revealed that depressed patients exhibited increased protein carbonylation in platelets, along with an overexpression of phosphodiesterase-5 and significantly heightened platelet aggregation. In another study by Williams et al. (51) involving 300 patients with cardiovascular disease comorbid with depression, it was observed that compared to patients with uncomplicated depression, those with comorbid depression had more serotonin receptors in their blood, and their platelets were more prone to aggregation. Similarly, a Chinese study also confirmed that patients with CHD combined with depression had elevated platelet specific volume and increased platelet activity (52). Therefore, based on these findings, it is plausible to hypothesize that platelet activation and aggregation may represent another essential mechanism through which depression contributes to the development of CHD.

Furthermore, 5-HT (serotonin) acts as a weak platelet activator, stored within platelets, and induces both platelet activation and coronary vasoconstriction. It also triggers other platelet activators, such as adenosine diphosphate, thromboxane A2, thrombin, and catecholamines, leading to increased platelet activity (53). In cases of depression, there is a reduction or abnormality in 5-HT levels, resulting in an up-regulation of 5-HT receptors on the platelet surface (53). This increased receptor responsiveness to 5-HT renders platelets more susceptible to activation. Moreover, depression can elevate the expression of integrin αIIb-β3 and increase plasma levels of platelet activation markers, such as platelet factor-4 (PF-4), β-thromboglobulin (β-TG), and P-selectin, ultimately enhancing platelet activity. A study demonstrated significantly higher levels of platelet activation markers, including PF-4 and β-TG, in patients with both CHD and depression compared to patients with CHD alone and healthy controls (54). Platelet activation not only fosters endothelial damage by interacting with leukocytes but also stimulates the release of related factors, further promoting the development of atherosclerosis. This has adverse implications for the prevention and treatment of CHD and heightens the risk of adverse cardiovascular events. Additionally, it has been suggested that depression-induced platelet aggregation may be related to the elevated levels of inflammatory factors induced by depression. These factors can promote the release of adhesion molecules from coronary endothelial cells (42), thereby exacerbating the progression of atherosclerosis and its detrimental impact on CHD management and cardiovascular outcomes.

### 3.6 Endothelial dysfunction

Depression’s impact on CHD is likely mediated through endothelial dysfunction. Endothelial dysfunction involves several key characteristics, such as reduced endothelium-dependent vasodilation and barrier function, hemodynamic abnormalities, impaired fibrinolytic capacity, increased expression of growth factors and adhesion molecules, and heightened oxidative stress, among others (55). Vascular endothelial dysfunction is the initiator of atherosclerosis, being a primary cause of diminished vascular reactivity and a significant pathomechanism in CHD. Depression can contribute to the progression of endothelial dysfunction, which, in turn, leads to the development and exacerbation of atherosclerosis. These harmful effects on the vascular endothelium can further compromise vascular health and increase the risk of CHD-related complications. Understanding the relationship between depression and endothelial dysfunction is crucial in comprehending the comprehensive mechanisms underlying the association between depression and CHD.

Flow-mediated dilatation (FMD) is a commonly used to assess vascular endothelial function. Patients with CHD combined with depression were found to have significantly lower absolute and percentage FMD compared to those with CHD alone, providing support for the notion that depression contributes to endothelial dysfunction (56). Furthermore, the correlation between endothelial dysfunction and depression persists even after adjusting for various influences, such as lifestyle factors (57). Nitric oxide (NO) is a critical mediator of vascular endothelial function and is a common vasodilator, which plays a crucial role in maintaining a healthy vascular system. NO promotes vasodilation, inhibits smooth muscle cell contraction, and prevents platelet aggregation and leukocyte adhesion. Reduced levels of endogenous NO can lead to endothelial dysfunction (58). Studies conducted by Greaney et al. (59) revealed that depressed patients experience increased oxidative stress in the vascular endothelium, along with decreased NO synthesis. Similarly, experimental studies on depressed rats have yielded similar findings. These rats exhibited significantly reduced endothelium-dependent vasodilatory responses, and direct administration of the NO donor sodium nitroprusside or the non-NO-dependent opioid agonist failed to induce vasodilation in the depressed rats (60). Severe depression has been associated with increased lipid peroxidation, elevated plasma levels of lipid peroxidation metabolites such as 4methyl-2-NONERAL and asymmetric dimethylarginine, decreased endothelial nitric oxide synthase (NOS) activity, and reduced plasma NO levels. These alterations impact vasoconstriction, neurotransmitter release, and perfusion, and contribute to endothelial damage. This cascade of events ultimately leads to platelet aggregation, an upsurge in inflammatory response, coronary artery constriction, and enhanced intercellular adhesion, significantly increasing CHD and cardiovascular events (25).

### 3.7 Disorders of lipid metabolism

Depression can lead to disorders of lipid metabolism, contributing to the development of CHD by promoting an increase in abdominal fat and epicardial fat surrounding the coronary arteries. Elevated lipids are well-known risk factors for CHD, particularly serum total cholesterol (TC) and low-density lipoprotein cholesterol (LDL-C). Clinical studies have shown that depression is associated with elevated TC and LDL-C levels, while triglyceride levels tend to be lower. Notably, the correlation between depression and LDL-C is more pronounced (61, 62). Animal studies have provided similar insights, demonstrating that simvastatin, a lipid-lowering drug commonly used in CHD, significantly improved depressive symptoms in mice with depression-like models (63). In addition to the overall lipid levels, oxidized LDL (ox-LDL) plays a crucial role in the pathogenesis of CHD. Maes et al. observed that depressed patients had higher levels of ox-LDL and lower levels of vitamin E compared to controls. These findings indicate that depressed patients have relatively enhanced lipid oxidation and weakened antioxidant protection mechanisms in their bodies, further predisposing them to CHD.

Abnormal metabolism of omega-3 polyunsaturated fatty acids is also a significant intermediate factor that affects the development of both depression and CHD. Omega-3 polyunsaturated fatty acids are important neurotransmitters, influencing depression by regulating 5-HT, dopamine, and brain-derived neurotrophic factor (64, 65). Evidence suggests that reduced levels of omega-3 polyunsaturated fatty acids are associated with the development of depression and other affective disorders in a bidirectional manner. Moreover, decreased levels of omega-3 polyunsaturated fatty acids are closely linked to the development of CHD. Under normal circumstances, these fatty acids exert beneficial effects by acting as anti-inflammatory agents, antioxidants, and regulators of lipid metabolism, thereby reducing the progression of atherosclerosis and stabilizing plaques. Studies have revealed that plasma levels of omega-3 polyunsaturated fatty acids in patients with CHD combined with depression are lower compared to those without comorbid depression. Notably, supplementation with omega-3 polyunsaturated fatty acids has been shown to slow the progression of CHD and decrease the risk of cardiovascular events (66). Hence, it is plausible that depression contributes to the development of CHD by affecting the metabolism of omega-3 polyunsaturated fatty acids. The interplay of these fatty acids in both depression and CHD underscores their significance in the pathogenesis and management of these conditions, emphasizing the importance of exploring their potential therapeutic implications in preventing and treating both depression and CHD.

### 3.8 Heredity

Depression, like traditional risk factors for CHD, has a hereditary component. However, direct evidence regarding the genetic mechanisms linking depression and CHD remains limited. In the early 21st century, a study involving 2,731 same-sex twins (mean age 41.9 years, SD 2.7) found a significant genetic correlation between depression, hypertension (r = 0.19), and CHD (r = 0.42). These findings suggest that common genetic risk factors may be involved in the association between depression and CHD (67). Subsequently, Lu et al. (68) conducted a Mendelian randomization and mediation analysis to evaluate the associations of depression-related genetic variants with CHD. They concluded that Genetic liability to depression is associated with higher CHD risks. These discoveries have led to the possibility that depression and CHD may represent different phenotypes of the same genes. However, some researchers, such as de Geus (69), argue that genetic pleiotropy does not rule out other possibilities. For instance, a particular gene may cause depression, and subsequently, depression may lead to or worsen CHD. This alternative explanation highlights the complex interplay of genetic and environmental factors in the relationship between depression and CHD. While the genetic basis of the association between depression and CHD warrants further investigation, it is evident that both conditions share common risk factors and biological pathways. The convergence of genetic, environmental, and behavioral factors likely contributes to the intricate relationship between depression and CHD. More comprehensive research is needed to unravel the underlying mechanisms and potential therapeutic implications for managing both depression and CHD effectively.

Nakatani et al. (67) examined the S allele of the 5-hydroxytryptamine transporter gene in 2,509 post-myocardial infarction patients. They found that individuals with the S allele had a higher prevalence of depression (48%) compared to those without it (35%). Furthermore, the presence of the S allele was associated with an increased risk of cardiovascular events (HR 1.69), but this effect became non-significant after adjusting for depressive symptoms. This suggests that depression has the potential to influence the prognosis of CHD. Subsequently, mounting evidence supports the notion that the 5-HT transporter (5-HTT) gene and the 5-HT receptor (5-HTR) gene may play a role in the process of depression contributing to CHD. Specifically, gene polymorphisms in the promoter sequence of the 5-HT transporter gene (5-HTTLPR) are closely associated with depression. The SS genotype of 5-HTTLPR restricts the transcriptional activity of the promoter, leading to a decrease in the level of transcription of the 5-HT transporter protein gene (SLC6A4) and a reduction in 5-HT reuptake, which can contribute to the development of depression (70). Furthermore, the SS genotype is also independently associated with comorbid depression in patients with CHD, making it a susceptibility gene for depression in individuals with CHD. Additionally, the haplotype angiotensin I-converting enzyme (ACE) gene D/D and 5-HTTLPR I/I may be a vulnerability factor for comorbid depressive symptoms in CHD patients (71). Therefore, it is hypothesized that the 5-HTTLPR gene polymorphism may represent one of the genetic mechanisms by which depression contributes to CHD. However, further in-depth research must validate and explore this hypothesis thoroughly.

### 3.9 Other emerging potential mechanisms

The study of microRNA (miRNA) alterations in CHD and depression is an evolving field, revealing the intricate role of miRNA in both systemic and mental health disorders. Numerous miRNAs exhibit altered expression levels during cardiovascular diseases such as atherosclerosis, myocardial infarction, and heart failure. Furthermore, miRNAs associated with various risk factors have contributed to the development of CHD (72, 73). MicroRNA-34a (miR-34a) is essential in regulating blood lipid, inflammation, cell adhesion molecules, and atherosclerosis; the latter factors are closely involved in coronary heart disease (CHD) etiology. MiR-34a was elevated in CHD patients compared to controls (p < 0.001), and it disclosed an excellent diagnostic value of CHD (74). Approximately 100 miRNAs involved in lipid metabolism have been identified, with miRNA-122 highlighted by Tsai et al. as capable of reducing the expression of genes involved in triglyceride metabolism through the adenosine monophosphate-activated protein kinase pathway (75).

Functional dysregulation of miRNA regulation has also been observed in depression (76, 77). MicroRNA-16, for instance, exerts antidepressant activity by inhibiting the expression of the serotonin transporter, reducing the reuptake of synaptic serotonin, and promoting serotonin signal transduction (78–80). Brain-derived neurotrophic factor (BDNF) plays a crucial role in modulating miRNA expression. BDNF, through the activation of the mitogen-activated protein kinase/extracellular signal-regulated kinase pathway, selectively upregulates the expression miRNA-132 in cultured rat cortical neurons. This upregulation correlates with increased axonal growth and dendritic spine numbers, suggesting that miR-132 may regulate BDNF-mediated neural plasticity (81–83). Considering the significant role of BDNF in the pathogenesis of depression, dysregulation of BDNF-associated miRNAs may also be closely linked to depression.

Research into miRNA changes in CHD and depression is rapidly advancing, underscoring the functional roles and dysfunctions of miRNA in both systemic and mental disorders. CHD and depression appear to share some common pathological mechanisms that depend on miRNA regulation. Although there is currently no evidence supporting the use of miRNA gene chip detection and related methods in patients with comorbid CHD and depression, these studies are in their early stages. Therefore, it is believed that miRNA will directly correlate with the comorbidity of CHD and depression shortly, becoming a valuable research tool and possibly a valuable therapeutic tool.

The gut microbiota is key in regulating physiological processes such as intestinal environmental stability, metabolic regulation, and immune responses. Recent studies have shown that the gut microbiota is closely related to the pathogenesis of coronary heart disease and depression, mainly through the multilevel regulation of the microbiota-gut-brain axis, encompassing inflammatory responses, chronic inflammation, and metabolites (84). Changes in the composition and diversity of the gut microbiota have been associated with the development of chronic inflammatory diseases, including cardiovascular disease. It has been noted that gut microbial disorders can trigger various pathophysiological events, including weight gain, adipose deposition, low-grade inflammatory response, and the association of reduced gut microbial diversity with the prevalence of diabetes mellitus, obesity, and metabolic syndrome (85). In addition, the gut microbiota is directly involved in the development of coronary atherosclerosis and may play a key role in cardiac metabolic homeostasis and cardiovascular disease through multiple pathways (86).

On the other hand, the gut microbiota composition may show differences in patients with CVD. Significant differences in the diversity and composition of the gut microbiota were found between patients with coronary artery disease and healthy controls, including a decrease in the phylum Bacteroidetes and Aspergillus and an increase in the phylum Fusobacterium and Fusobacterium (87, 88). The presence of Aspergillus bacteria in the bloodstream has been strongly correlated with cardiovascular complications and may serve as an independent risk marker for cardiovascular disease (89, 90).

Clinical studies have confirmed that changes in the microbiota characterization are strongly associated with an increased risk of cardiovascular disease. Different abundances of the microbiota may be more important in determining the risk of cardiovascular disease than their relative frequencies. In addition, the composition and relative abundance of the gut microbiota impact neurologic function, suggesting a strong link between cardiovascular and neurologic diseases (91). The gut microbiota has been shown to impact neurologic function significantly.

The gut microbiota influences brain development and function by releasing metabolites or stimulating the production of neuroactive substances by endocrine cells in the gastrointestinal tract (92–94). The brain monitors and regulates the composition of the gut microbiota through neural, immune, and endocrine pathways to maintain a normal species profile or adjust the microbial profile in response to environmental changes (95–97). The beneficial role of probiotics in depressed patients may be related to vagal regulatory pathways (98). However, the “microbiota-gut-brain axis” interactions may also lead to alterations in the microbial environment, which may affect neural activity and function and trigger pathological outcomes such as depression (87). Clinical studies have demonstrated that patients with depression have changes in the composition and relative abundance of gut flora, which are mediated through inflammatory responses, HPA, and other factors that may affect the gut flora’s ability to respond to the environment. Changes induce depression by altering CNS function through inflammatory responses, HPA axis activity, and neurotransmitter signaling (99, 100). Therefore, restoration of physiological homeostasis of the CNS and modulation of inflammatory factors and neurotransmitters by adjusting the composition and function of gut microflora may improve depressive behavior.

## 4 Interventions

The United States Preventive Services Task Force (USPSTF) once emphasized the increased risk of depression in patients with cardiovascular diseases (101). However, despite the gradual increase in depression screening since 2009, only 3% of outpatients still received depression screening by 2015 (102). Although providing reasonable intervention methods for CHD patients with depression is particularly important, accurate screening and diagnosis are prerequisites. Therefore, developing standardized screening methods for depression in CHD patients provides potential for early recognition and prevention of CHD and depression (103).

Current clinical treatment options for CHD co-morbid with depression encompass medication, psychological intervention therapy, exercise therapy, and acupuncture. Antidepressant treatment, combined with conventional CHD therapy, has improved psychological status and symptoms (8). Selective 5-hydroxytryptamine reuptake inhibitors (SSRIs) are the preferred antidepressants due to their anti-inflammatory, antiplatelet aggregation, and HPA axis activity reduction effects (104). These SSRIs increase the concentration of synaptic 5-HT, which helps inhibit depression-induced CHD with minimal side effects on the cardiovascular system. Sertraline hydrochloride, paroxetine, and citalopram are among the commonly used SSRIs. Notably, sertraline has significantly reduced the recurrence of myocardial infarction (MI) and mortality, making it the first-line drug for treating depression combined with CHD (105). Studies have also suggested that sertraline treatment may improve vascular endothelial function and reduce adverse cardiac events by decreasing platelet activity (106). However, evidence from further studies is required to corroborate this. Other SSRIs, such as citalopram and fluoxetine, researches have demonstrated positive effects on reducing myocardial necrosis and improving prognosis (107, 108). However, animal experiments have yielded varying conclusions regarding the effect of antidepressant treatment on CHD, warranting further investigation. It’s essential to be cautious about potential drug interactions when prescribing SSRIs to patients concurrently taking other CHD medications, as some SSRIs are metabolized by the same hepatic enzyme (P450).

Unlike Western medicine treatment, traditional Chinese medicine (TCM) for treating depression with CHD is based on syndrome differentiation and treatment, with the advantages of multi-target, multi-pathway, overall treatment, and security good (109). Chen et al. (110) found through meta-analysis that, a well-known Chinese patent drug, Xinkeshu tablets (XKS), might benefit CHD patients experiencing depression after PCI by helping to improve their depression symptoms, TC and TG blood lipid levels. A meta-analysis and systematic review by Wang et al. (109) included 32 studies, which results showed that compared with the blank control groups, TCM was more beneficial in treating depression in patients with CHD [depression: OR = 3.27, 95% CI (1.67, 6.40), p = 0.0005, I2 = 0%], and the efficacy of TCM was not inferior to that of Western medicine [depression: OR = 1.97, 95%CI (0.73, 5.28), p = 0.18, I2 = 33%],. In addition, TCM also showed a significant advantage in improving the symptoms of angina pectoris in CHD patients with depression. And the most closely and simultaneously related to the pathological targets of CHD and depression, the most active compounds of TCM, such as quercetin, kaempferol, puerarin, baicalein, tanshinone IIa, mainly exert their effects through anti-inflammatory, antioxidant stress, and anti-damage/apoptosis underlying pathways. On the other hand, experimental studies have found that acupuncture, as one of the important therapies of traditional Chinese medicine, can significantly increase the serum levels of Glutathione and Catalase, the expression of Beclin-1 and LC3B proteins in the hippocampus of rats with depression and CHD. This therapeutic effect may be closely related to mitochondrial autophagy and oxidative stress (111, 112). From the perspective of combining Western medicine and traditional Chinese medicine in the future, providing antidepressant treatment for CHD patients with depression can be an effective and safe approach. **Table 1** presents the medication details of antidepressant treatment for CHD with depression.

**Table 1:** Antidepressant treatment for CHD with depression.

Drug	Daily Dose	Cardiovascular Side-effects	Drug Interactions
**SSRIs**		The risk of bleeding is increased.	Patients taking anticoagulants or aspirin should be used with caution, and thiazide diuretics may cause severe hyponatremia.
Sertraline	Initial dose: 50 mg Target dose: 50-200 mg	Palpitations, tachycardia.	Prohibition of combination with MAOIs and Pimozide.
Paroxetine	Initial dose: 20 mg^*^; 25 mg^**^ Target dose: 20-50mg^*^; 25-62.5mg^**^	Transient blood pressure changes.	Prohibition of combination with MAOIs, and combined use with warfarin may cause increased bleeding.
Citalopram	Initial dose: 20 mg Target dose: 20-40 mg	Tachycardia, QTc prolongation, hypotension, postural hypotension.	Decreased metabolism of β-blockers and certain anti-arrhythmic drugs, and when used in combination with Droperidol, cardiac toxicity may increase.
Escitalopram	Initial dose: 10 mg Target: dose: 10-20 mg	Palpitations, hypertension.	Decreased metabolism of β-blockers and certain anti-arrhythmic drugs.
Fluoxetine	Initial dose: 20 mg Target dose: 20-80 mg	Vasodilatation.	Decreased metabolism of β-blockers and certain anti-arrhythmic drugs.
**SNRIs**		Hypertension, tachycardia, and the risk of bleeding are increased.	Drug Interactions are the same as SSRIs.
Levomilnacipran	Initial dose: 40 mg Target dose: 40-120 mg	Increased heart rate.	Strong CYP3A4 inhibitors (such as ketoconazole) can significantly increase the plasma concentration of levomilnacipran.
Venlafaxine	Initial dose: 75 mg Target dose: 150-375 mg	Sustained hypertension at doses > 300 mg/day, cholesterol elevation, and orthostatic hypotension.	Combined use with warfarin may cause increased bleeding.
Desvenlafaxine	Initial dose: 50 mg Target dose: 100 mg	Orthostatic hypotension in patients≥65 years old, cholesterol and triglyceride elevation	/
Others			
Vortioxetine	Initial dose: 10 mg Target dose: 10 mg	The risk of bleeding is increased.	Prohibition of combination with non-selective MAOIs or selective MAO-A inhibitors.
Vilazodone	Initial dose: 10 mg Target dose: 40 mg	The risk of abnormal bleeding when used in combination with NSAIDs, aspirin, and warfarin is increased.	Prohibition of simultaneous use with MAOIs.
Mirtazapine	Initial dose: 15 mg Target dose: 15-45 mg	Hypertension, orthostatic hypotension.	Be cautious of simultaneous use with MAOIs. Enhanced the activity of warfarin and potential of thiazide diuretics in the treatment of hyponatremia.
Olanzapine	Initial dose: 5 mg Target dose: 5-20 mg	Postural hypotension, hypertension, QTc prolongation, tachycardia.	Enhanced effect of antihypertensive drugs.
Trazodone	Initial dose: 150 mg Target dose: 150-375 mg^***^; 300-500 mg^*^	The risk of bleeding is increased, orthostatic hypotension, QTc prolongation.	Prohibition of simultaneous use with MAOIs, and use with caution in patients on anticoagulants or aspirin.
Quetiapine	Initial dose: 50 mg Target dose: 150-300 mg	Hyperglycemia, hyperlipidemia, tachycardia, hypertension, orthostatic hypotension.	Enhanced effect of antihypertensive drugs.
Aripiprazole	Initial dose: 2.5 mg Target dose: 5-15 mg	Palpitations, hypotension, bradycardia, QTc prolongation.	Enhanced effect of antihypertensive drugs by α- adrenergic antagonism.

CHD, Coronary heart disease; SSRIs, selective serotonin reuptake inhibitor; SNRIs, serotonin-norepinephrine reuptake inhibitor; MAOIs, monoamine oxidase inhibitors; MAO-A, monoamine oxidase-A; NSAIDs, non-steroidal anti-inflammatory drugs; *immediate release; **controlled release; ***extended release.

Psychological interventions, such as cognitive-behavioral therapy, interpersonal psychotherapy, and problem-solving therapy, have shown promise in reducing depressive symptoms, improving treatment compliance, and preventing relapse when combined with medication (113). These interventions have led to gradual improvements in the condition of CHD patients (114). However, while behavioral cognitive therapy has been associated with improved psychological status and quality of life, it does not significantly impact cardiac-related mortality (115). Additionally, psychological interventions have shown benefits in reducing cardiac deaths and alleviating psychological symptoms compared to conventional treatment, but they do not reduce overall mortality (111). Despite the lack of reduced endpoint events, antidepressant treatment has been shown to alleviate depressed mood and improve the quality of life in patients, making it a valuable therapeutic approach for CHD patients with comorbid depression. Exercise therapy, tailored to individual cardiac function and exercise endurance, can also play a role in reducing depression and CHD-related symptoms (115). This treatment modulates inflammatory factors and the parasympathetic nervous system, effectively reducing prevalence and mortality in co-morbid patients (115). Encouraging lifestyle changes can further support this approach. A comprehensive treatment approach that combines medication, psychological interventions, and exercise therapy should be actively administered to CHD patients with comorbid depression.

The relationship between depression and CHD is a significant concern, and it is crucial to determine whether combining antidepressant therapy with standard CHD treatment can improve symptoms and prognosis. Studies have shown that antidepressant treatment for patients with CHD and depression leads to substantial improvements in chest pain symptoms, depressive symptoms, and various cardiac parameters (109, 116, 117). Furthermore, effective antidepressant treatment in depressed patients after acute myocardial infarction (AMI) has been associated with reduced adverse cardiovascular events and improved survival during a 29-month follow-up (118). Interventions to address depressive symptoms in patients with acute coronary syndrome (ACS) have also proven effective in reducing the number of ACS-related re-hospitalizations. However, this benefit may not be sustained after treatment discontinuation (119). However, conflicting results have been reported, with some studies indicating that the benefits of antidepressant treatment for CHD are not significant (120–122). This suggests that although cognitive behavior therapy can alleviate depressive symptoms in CHD patients, it is only during short-term follow-up, and its impact on the long-term prognosis is not definite (120). Meanwhile, for patients with MI and depression, antidepressant treatment does not seem to reduce long-term major adverse cardiovascular events (MACE) or all-cause mortality (121). Similarly, Tully et al. concluded that while antidepressant treatment may lower the incidence of short-term MACE in CHD patients with depression, its impact on medium- and long-term MACE rates appears insignificant (123). Given the mixed findings, more research is needed to ascertain the true efficacy of antidepressant therapy in improving the prognosis of CHD patients with comorbid depression. Future studies should consider larger sample sizes, longer follow-up periods, and potentially tailored treatment approaches based on individual patient characteristics to determine the most effective course of action. Additionally, understanding the potential factors contributing to the varying results can help develop targeted interventions for specific subgroups of patients with CHD and depression.

Researchers have increasingly recognized the combination of multiple interventions for patients with comorbid CHD and depression. Studies have shown that screening CHD patients for depression and providing self-management instructions for depressive symptoms can improve clinical outcomes (122). Guidelines recommend various interventions for CHD patients with depression, including stress management, individual or group counseling, self-care practices, and supportive pharmacotherapy (22). For patients with depression and significant symptoms or psychosocial factors, a combination of interventions from different channels is often more effective, such as psychotherapy, pharmacotherapy, and collaborative care. Cardiovascular specialists need to be vigilant about screening for depressive symptoms in CHD patients to enhance clinical care and improve their quality of life. When administering antidepressant therapy to depressed patients with comorbid CHD, psychologists should comprehensively assess the potential effects of antidepressant medications on cardiovascular disease. In cases where a more holistic approach is necessary, a multidisciplinary approach can be adopted to develop the best treatment plan for the patients. By incorporating various interventions and considering each patient’s unique needs, healthcare professionals can effectively address both the cardiovascular and mental health aspects of comorbid CHD and depression, leading to better overall patient outcomes.

The interrelationship between depression and CHD has garnered significant global. However, there remains a lack of expert consensus and authoritative guidelines on clinical interventions for patients with these co-morbidities. Several reasons contribute to this situation:

1. Contradictory Conclusions: There are still divergent findings regarding the relationship between depression and CHD, and an international consensus on the precise pathogenesis of depression’s impact on CHD is yet to be established. This lack of clarity makes it challenging to develop targeted interventions based on the underlying mechanisms.
2. Complex Diagnosis and Treatment: Assessing psychiatric and psychological factors in clinical diagnosis and treatment requires specialized expertise, which can be intricate. This complexity presents challenges in comprehensive interventions for patients with comorbid depression and CHD.
3. Unclear Impact of Antidepressant Treatment: The effect of antidepressant treatment on CHD is not fully understood, and there are no definitive treatment guidelines to guide clinicians in managing patients with both conditions. Addressing these issues will necessitate further research and exploration in the future. Efforts should be made to understand the relationship between depression and CHD better, develop effective treatment guidelines, and improve the clinical management of patients with these co-morbidities. With continued investigation and collaboration, we can work towards providing more comprehensive and targeted care for individuals affected by both depression and CHD.

## 5 Conclusions

Currently, most of the existing literature on CHD co-morbid with depression focuses on observational studies, with limited research on the physiological mechanisms that underlie the impact of depression on CHD and potential therapeutic interventions. The identified physiological mechanisms linking depression and CHD primarily revolve around inflammatory response, HPA axis dysfunction, autonomic dysfunction, endothelial dysfunction, and platelet activation. However, there is still much to explore concerning mechanisms like D-type personality and genetics. Although it is generally agreed upon that combining pharmacological and non-pharmacological treatments offers the best approach for managing CHD and depression, further comprehensive experimental and clinical studies are essential to validate its efficacy and safety. Additionally, developing treatment guidelines for managing these co-morbidities requires greater attention. In addition to the existing knowledge, it is important to comment on future research and clinical practice prospects. Moving forward, a more integrated and interdisciplinary approach is needed to unravel the complex interplay between depression and CHD. This could involve exploring novel mechanisms such as the role of epigenetic factors, immune dysregulation, and the gut-brain axis. Additionally, leveraging technological advancements, such as wearable devices and mobile health applications, could aid in monitoring and managing the co-morbidities more effectively. Furthermore, identifying specific subgroups of patients who are more vulnerable to the detrimental effects of depression on CHD could help tailor interventions and improve patient outcomes. To advance our understanding and treatment of CHD combined with depression, it is crucial to conduct more in-depth investigations to identify the primary mechanisms and their interactions. This will enable more effective and safe interventions, providing patients with a broader range of treatment options and ultimately enhancing their overall quality of life. By dedicating efforts to research further, we can improve care for patients with CHD and depression.

## Author contributions

LX: Conceptualization, Writing – original draft. XZ: Formal analysis, Writing – review & editing. DS: Conceptualization, Writing – original draft. YZ: Methodology, Writing – review & editing.
